# Associations between anxiety, sleep, and blood pressure parameters in pregnancy: a prospective pilot cohort study

**DOI:** 10.1186/s12884-024-06540-w

**Published:** 2024-05-15

**Authors:** Hayley E. Miller, Samantha L. Simpson, Janet Hurtado, Ana Boncompagni, Jane Chueh, Chi-Hung Shu, Fiona Barwick, Stephanie A. Leonard, Brendan Carvalho, Pervez Sultan, Nima Aghaeepour, Maurice Druzin, Danielle M. Panelli

**Affiliations:** 1 Department of Obstetrics and Gynecology, Division of Maternal-Fetal Medicine and Obstetrics, Stanford University School of Medicine, 453 Quarry Road, Stanford, Palo Alto, CA 94304 USA; 2 Stanford School of Medicine, Stanford, CA USA; 3 Department of Anesthesiology, Division of Obstetric Anesthesiology and Maternal Health, Perioperative and Pain Medicine, Stanford University School of Medicine, Stanford, CA USA; 4 Department of Psychiatry and Behavioral Sciences, Division of Sleep Medicine, Stanford University School of Medicine, Stanford, CA USA

**Keywords:** Anxiety, Insomnia, Short sleep duration, Blood pressure, Hypertension, Pregnancy, Actigraphy, Systolic blood pressure, Diastolic blood pressure, Mean arterial blood pressure, Stress

## Abstract

**Background:**

The potential effect modification of sleep on the relationship between anxiety and elevated blood pressure (BP) in pregnancy is understudied. We evaluated the relationship between anxiety, insomnia, and short sleep duration, as well as any interaction effects between these variables, on BP during pregnancy.

**Methods:**

This was a prospective pilot cohort of pregnant people between 23 to 36 weeks’ gestation at a single institution between 2021 and 2022. Standardized questionnaires were used to measure clinical insomnia and anxiety. Objective sleep duration was measured using a wrist-worn actigraphy device. Primary outcomes were systolic (SBP), diastolic (DBP), and mean (MAP) non-invasive BP measurements. Separate sequential multivariable linear regression models fit with generalized estimating equations (GEE) were used to separately assess associations between anxiety (independent variable) and each BP parameter (dependent variables), after adjusting for potential confounders (Model 1). Additional analyses were conducted adding insomnia and the interaction between anxiety and insomnia as independent variables (Model 2), and adding short sleep duration and the interaction between anxiety and short sleep duration as independent variables (Model 3), to evaluate any moderating effects on BP parameters.

**Results:**

Among the 60 participants who completed the study, 15 (25%) screened positive for anxiety, 11 (18%) had subjective insomnia, and 34 (59%) had objective short sleep duration. In Model 1, increased anxiety was not associated with increases in any BP parameters. When subjective insomnia was included in Model 2, increased DBP and MAP was significantly associated with anxiety (DBP: β 6.1, *p* = 0.01, MAP: β 6.2 *p* < 0.01). When short sleep was included in Model 3, all BP parameters were significantly associated with anxiety (SBP: β 9.6, *p* = 0.01, DBP: β 8.1, *p* < 0.001, and MAP: β 8.8, *p* < 0.001). No moderating effects were detected between insomnia and anxiety (p interactions: SBP 0.80, DBP 0.60, MAP 0.32) or between short sleep duration and anxiety (p interactions: SBP 0.12, DBP 0.24, MAP 0.13) on BP.

**Conclusions:**

When including either subjective insomnia or objective short sleep duration, pregnant people with anxiety had 5.1–9.6 mmHg higher SBP, 6.1–8.1 mmHg higher DBP, and 6.2–8.8 mmHg higher MAP than people without anxiety.

## Background

Hypertensive disorders occur in approximately 10% of all pregnancies in the United States [1]. Elevated blood pressure in pregnancy poses maternal and fetal risks, including preterm birth, placental abruption, cerebral hemorrhage, hepatic failure, and acute renal failure. Societies including American College of Obstetricians and Gynecologists (ACOG), Society for Maternal–Fetal Medicine, and California Maternal Quality Care Collaborative focus on mitigating these risks by implementing guidelines and toolkits to reduce the incidence of hypertensive disorders of pregnancy and treat elevated blood pressure appropriately [1–3]. Mood disorders and sleep disturbances are known modifiable risk factors for hypertension [4–16]. However, the degree to which these conditions affect specific blood pressure parameters in pregnancy is less well described in the literature. There are limited data on the specific impact of these conditions on specific blood pressure parameters in pregnancy. Understanding and addressing modifiable risk factors for elevated blood pressure in pregnancy may improve maternal and fetal outcomes and can be implemented as a public health priority.

Sleep disturbances such as difficulty falling asleep, increased nighttime awakenings, and shorter total sleep time are more common in pregnant people compared to the general population, with an incidence ranging between 46–78% [17]. Clinical insomnia is suggested using validated patient-reported questionnaires such as the Insomnia Severity Index (ISI), which rely on subjective assessment of a person’s sleep. Clinical insomnia is characterized by the subjective perception of sleep disturbance, including problems falling asleep and staying asleep, and it can be assessed by validated self-report measures such as the Insomnia Severity Index. It has been associated with adverse perinatal outcomes, including preterm birth and elevated blood pressure [4–16, 18, 19]. Objective short sleep duration is defined as the total number hours of sleep and can be measured using physiological data as recorded by actigraphy, which is a wrist-worn triaxial accelerometer. Sleep duration measured objectively, and clinical insomnia measured subjectively can be distinct phenomena, as evidenced by the common discrepancy between these two methods of assessment [20, 21]. Like subjective insomnia, objective short sleep duration has also been associated with adverse outcomes, including hypertension, cardiovascular disease and neurocognitive impairment [20–23]. Sleep abnormalities such as clinical insomnia and objective short sleep duration can have a bidirectional relationship with potentiating effects on anxiety [24].

Similar to insomnia, clinical anxiety is linked to increased risk of hypertensive disorders of pregnancy and is also prevalent in pregnancy, with an overall incidence of 15%, and is also associated with multiple adverse birth outcomes [11]. The relationship between anxiety and hypertensive disorders of pregnancy is complex and not well studied, with several proposed biologically plausible pathways, including alterations to inflammatory, autonomic, hypothalamic–pituitary–adrenal activity physiologic processes [12–15, 24].

Despite the overlap between clinical insomnia, objective short sleep duration, and anxiety impacts on the risk of hypertensive disorders of pregnancy, little is known about how the effects of these conditions on specific blood pressure parameters. Our aims were to evaluate the relationship between anxiety, insomnia, and short sleep duration, as well as any moderating effects between these variables, on blood pressure during pregnancy. We hypothesized that anxiety would increase all blood pressure parameters, and that insomnia and short sleep duration would have an additive effect when co-occurring with anxiety.

## Methods

This was a pilot prospective cohort study of pregnant people between ages 22 and 42 at a single academic institution between November 2021 and July 2022. Participants were between 23 to 36 weeks’ gestation with a viable singleton or multiple pregnancy without life-limiting fetal anomalies and with planned delivery at Lucile Packard Children’s Hospital (LPCH), Stanford. Participants were matched by gestational age by inpatients and outpatients within two weeks of gestation. Participants were screened for eligibility using electronic medical records in the LPCH antepartum unit and obstetrics clinics. Only participants who confirmed their interest to participate in research by LPCH screening were approached for enrollment. Participants were excluded if they had an indication for delivery within the next seven days at the time of eligibility screening, had an allergy to rubber or steel, were unable or unwilling to remove other activity monitors during the study period, had been placed on bedrest, or had implanted electronic medical devices. Study participation lasted seven days. This study was approved by the Stanford Institutional Review Board (IRB Protocol 59752) and all participants confirmed and signed informed consent.

Study participants completed a baseline questionnaire that included demographic, health, and socioeconomic information, as well as questions related to mental health, exercise, and sleep. At the time of enrollment, participants completed validated self-report questionnaires of insomnia (ISI) and anxiety [State-Trait Anxiety Inventory (STAI)] [25, 26]. The ISI is a 7-item measure assessing the frequency and severity of both nighttime and daytime symptoms of insomnia. The range of scores is between 0 and 28. Our study used a cut-off score ≥ 15 to identify moderate to severe insomnia symptoms [25]. The STAI consists of 40 items measured on a 4-point Likert scale, with subscales reflecting separate components of state versus trait anxiety [24]. We defined clinical anxiety by STAI-State (STAI-S) score ≥ 40 [26].

Separately, to measure objective short sleep duration, study participants wore an ActiGraph watch (ActiGraph Corp, Pensacola, FL) for one week, a commercially available and FDA 510(k)-cleared Class II medical device. To be included, participants were required to wear the watch for at least 24 h and up to seven days. Participants were contacted by research coordinators or registered nurses at least two times per week on day three and day seven during the study to confirm compliance with study procedures. Downloaded 60-s epoch AGD files were processed using ActiGraph Software (version 6.13.4). Total sleep duration in minutes, defined as the period from estimated sleep onset to estimated sleep offset, was extracted from the Actigraph device. Our study used a sleep duration < 5 h as the marker for objective short sleep duration based on previous data suggesting adverse health and pregnancy outcomes associated with total sleep duration < 5 h [27–29]. We used the average of total sleep time per day throughout the study duration to classify a participant as short sleep duration. The 7-day study timeline is also consistent with or longer than prior studies using ActiGraph watches, some of which only include 1–3 days of sleep data [30–32]. Participants who delivered < 24 h after initiation of the ActiGraph watch were excluded from analyses.

Sociodemographic and clinical characteristics of study participants that were not otherwise collected from study questionnaires were extracted from electronic health records. Perinatal mental health and hypertensive disorders of pregnancy are influenced by sociodemographic characteristics and social determinants of health, including maternal age, insurance status, and marital status [33]. Race and ethnicity were included given the adverse effects that structural racism and discrimination have been shown to have on perinatal health. The category “other/more than one race” was used for pregnant people who did not self-identify with one of the pre-specified categories, and the category “unknown” was used for those who chose not to report their status. Clinical characteristics abstracted included any medical history (hypertension, antihypertensive use, pre-existing diabetes, kidney disease, epilepsy, pre-existing anxiety or mood disorders, autoimmune disease, infectious diseases), parity, body mass index (BMI; kg/m^2^) at time of enrollment, medication use in current pregnancy, and history of preeclampsia in previous pregnancies.

The independent variables were anxiety and either insomnia or short sleep duration, as well as the interaction between anxiety and insomnia or sleep duration. The dependent variables of the study were systolic blood pressure (SBP), diastolic blood pressure (DBP), and mean arterial pressure (MAP). All blood pressure parameters were measured using a Philips Model non-invasive blood pressure device and collected upon enrollment into the study as a one-time measurement.

### Statistical analysis

Baseline sociodemographic and clinical characteristics were compared between participants with and without anxiety using Student’s t-test, Wilcoxon rank sum test, or Fisher’s exact test where appropriate. Separate sequential linear regression models were conducted and we fit the models with generalized estimating equations (GEE) to account for matching by gestational age. First, the base model (Model 1) evaluated the association between anxiety (independent variable) and each BP parameter (dependent variable), adjusting for age, BMI, and antihypertensive medications as potential confounders. Next, Model 2 was conducted replicating Model 1 plus clinical insomnia and an interaction term between insomnia and anxiety as covariates. Lastly, Model 3 was conducted replicating Model 1 plus short sleep duration and an interaction term between short sleep and anxiety as covariates. Models were done with an identity link and a normal distribution accounting for gestational age matching within the cohort because participants were recruited from different clinical settings including high-risk and low-risk outpatients and admitted inpatients.

## Results

**Fig. 1:**
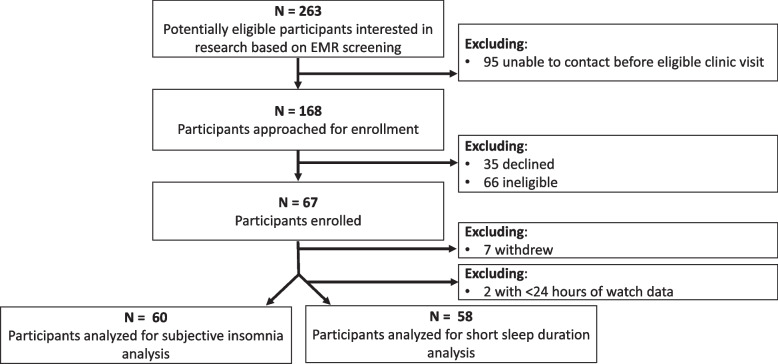
Study population

Among 263 participants who were eligible based on electronic medical record screening, 168 were approached for enrollment. Of those approached, 67 participants were enrolled. Among those, 7 withdrew from the study and 2 withdrew less than 24 h after enrollment, leaving 60 participants included in the analysis using self-reported insomnia data and 58 participants using ActiGraphic sleep data (Fig. 1). Baseline sociodemographic characteristics between those with and without clinical levels of state-anxiety symptoms were not significantly different (Table 1).

**Table 1:** Baseline characteristics among pregnant participants compared between those with versus without clinical anxiety

Baseline Characteristics	No anxiety *N* = 45	Clinical anxiety *N* = 15	*p*-value
	Mean ± SD	Mean ± SD	
Maternal age (years)	33.4 ± 4.8	33.2 ± 5.5	0.93
Gestational age (weeks)	30.8 ± 9.2	29.7 ± 3.7	0.84
	n (%)	n (%)	
Race			
Asian	1 (2.2)	0	0.90
Black	11 (24.4)	3 (20.0)	
Other/More than one race	15 (33.3)	7 (46.7)	
Native Hawaiian/Pacific Islander	2 (4.4)	1 (6.7)	
Unknown/Not reported	15 (33.3)	4 (26.7)	
White	1 (2.2)	0	
Ethnicity			
Hispanic or Latino	15 (33.3)	7 (46.7)	0.35
Non-Hispanic or Latino	27 (60.0)	6 (40.0)	
Unknown/Not reported	3 (6.67	2 (13.3)	
Body mass index (kg/m^2^)			
< 25	15 (33.3)	4 (26.7)	0.43
25–34	22 (48.9)	6 (40.0)	
≥ 35	8 (17.8)	5 (33.3)	
Nulliparous	24 (53.3)	8 (53.3)	1.00
Insurance Status			
Private	33 (73.3)	10 (66.7)	0.64
Public	11 (24.4)	5 (33.3)	
Other	1 (2.2)	0	
Total watch-worn days	6.5 (1.4)	6.4 (1.3)	0.39
Clinical insomnia (ISI > 15)	5 (11.1)	6 (40.0)	0.02
Objective short sleep duration	24 (53.3)	10 (29.4)	0.22
Antihypertensive use	3 (6.7)	1 (6.7)	1.0

Clinical anxiety defined by a State Trait Anxiety Inventory Score ≥ 40

*ISI* Insomnia Severity Index, *MAP* mean arterial blood pressure, *SBP* systolic blood pressure, *DBP* diastolic blood pressure, *M* mean, *SD* standard deviation

Of note, our cohort included 6 people with chronic hypertension, 3 with gestational hypertension, and 2 with preeclampsia (one with and one without severe features).

Among the 60 participants, 15 (25%) had clinical anxiety, 11 (18%) had clinical insomnia, and 34 (59%) had objective short sleep duration. A total of 6 (10%) had concurrent anxiety and clinical insomnia, while 10 (17%) had concurrent anxiety and short sleep duration. Clinical insomnia and anxiety were significantly associated (*p* = 0.02), but objective short sleep duration and anxiety were not (*p* = 0.22) (Table 1). Among those with clinical insomnia, the mean ISI score was 17.5 ± 3.3. Among those with short sleep duration the mean total sleep time was 233.5 ± 33.7 min and among those with anxiety, the mean STAI-S score was 48.9 ± 6.7.

**Fig. 2:**
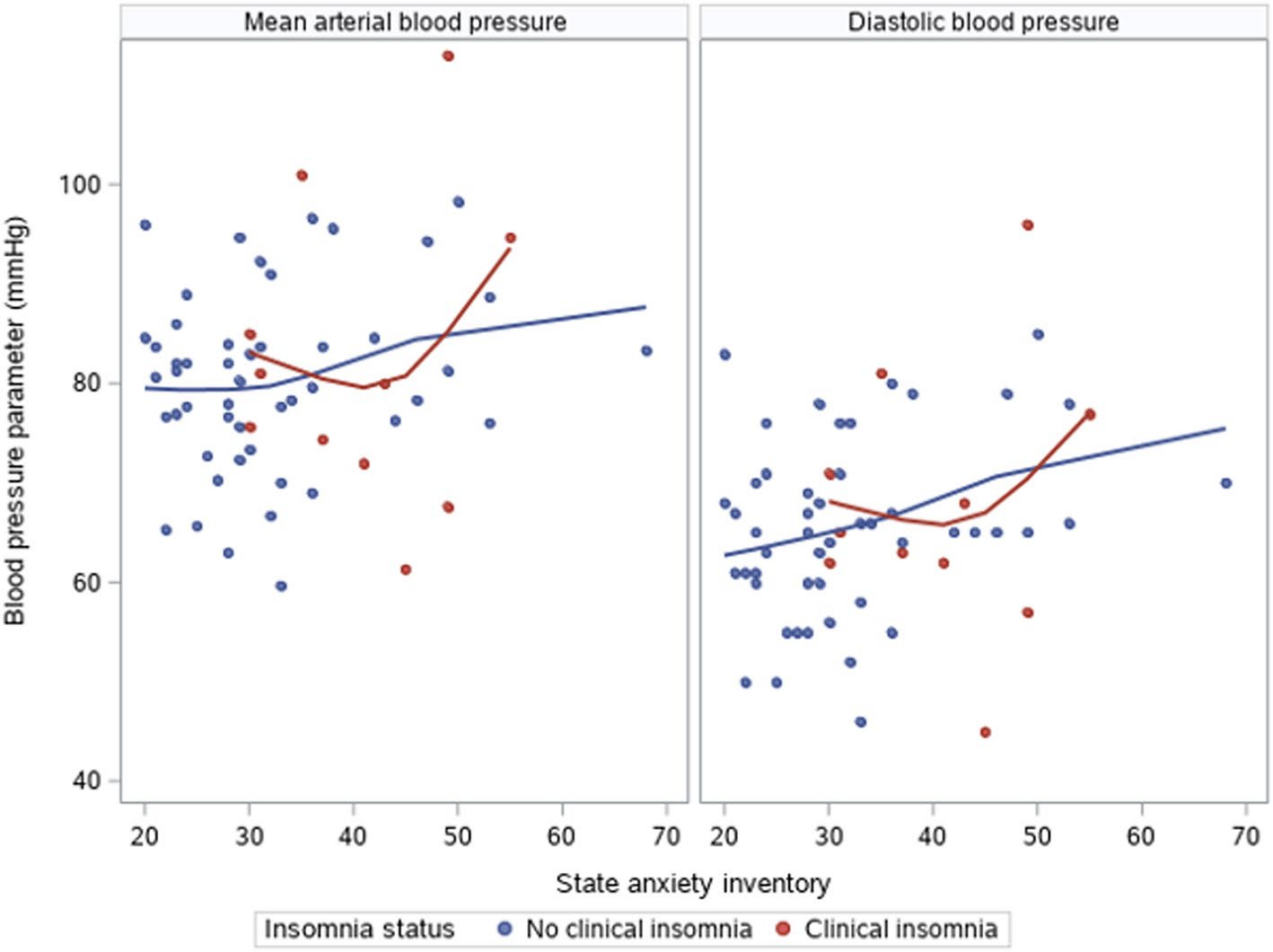
State anxiety inventory score and blood pressure parameter associations stratified by clinical insomnia.  Clinical insomnia defined as Insomnia Severity Index ≥ 15 SBP is not shown as it was not statistically significant

Model 1 demonstrated no baseline association between anxiety and any BP parameter after adjusting for demographic covariates and use of antihypertensive medications (SBP: β 1.3, *p* = 0.67, DBP: β 4.6, *p* = 0.07, MAP: β 3.6, *p* = 0.15). In Model 2, after additionally adjusting for clinical insomnia (based on ISI score), anxiety was significantly associated with increased DBP (β 6.1, *p* < 0.01) and MAP (β 6.2, *p* < 0.01) but not SBP (β 5.1, *p* = 0.07). There was no significant moderating effect between clinical insomnia and anxiety on BP parameters (p-interaction SBP 0.80, DBP 0.60, and MAP 0.32) (Table 2, Fig. 2).

**Table 2:** Association between anxiety and blood pressure parameters

Blood pressure parameter (mmHg)	**Model 1**^a^ β (95% CI) ***N* = 60**	***p*-value**	**Model 2**^b^ β (95% CI) ***N* = 60**	***p*-value**	**Model 3**^c^ β (95% CI) ***N* = 58**	***p*-value**
Systolic blood pressure	1.3 (-4.7, 7.3)	0.67	5.1 (-0.3, 10.4)	0.07	9.6 (1.9, 17.4)	0.01
Diastolic blood pressure	4.6 (-0.4, 9.6)	0.07	6.1 (1.9, 10.3)	0.01	8.1 (4.2, 12.0)	< 0.001
Mean arterial pressure	3.6 (-1.3, 8.5)	0.15	6.2 (1.9, 10.4)	0.004	8.8 (4.3, 13.4)	< 0.001

Clinical insomnia defined as Insomnia Severity Index ≥ 15 and objective short sleep duration defined as total sleep time < 5 h

^a^Model 1 is a linear regression model fit with generalized estimating equations. Covariates included age, body-mass index, and use of antihypertensive medications

^b^Model 2 replicates Model 1, adding clinical insomnia and an interaction term for insomnia and anxiety as a covariates

^c^Model 3 replicates Model 1, adding short sleep duration and an interaction term for short sleep duration and anxiety as a covariates

**Fig. 3:**
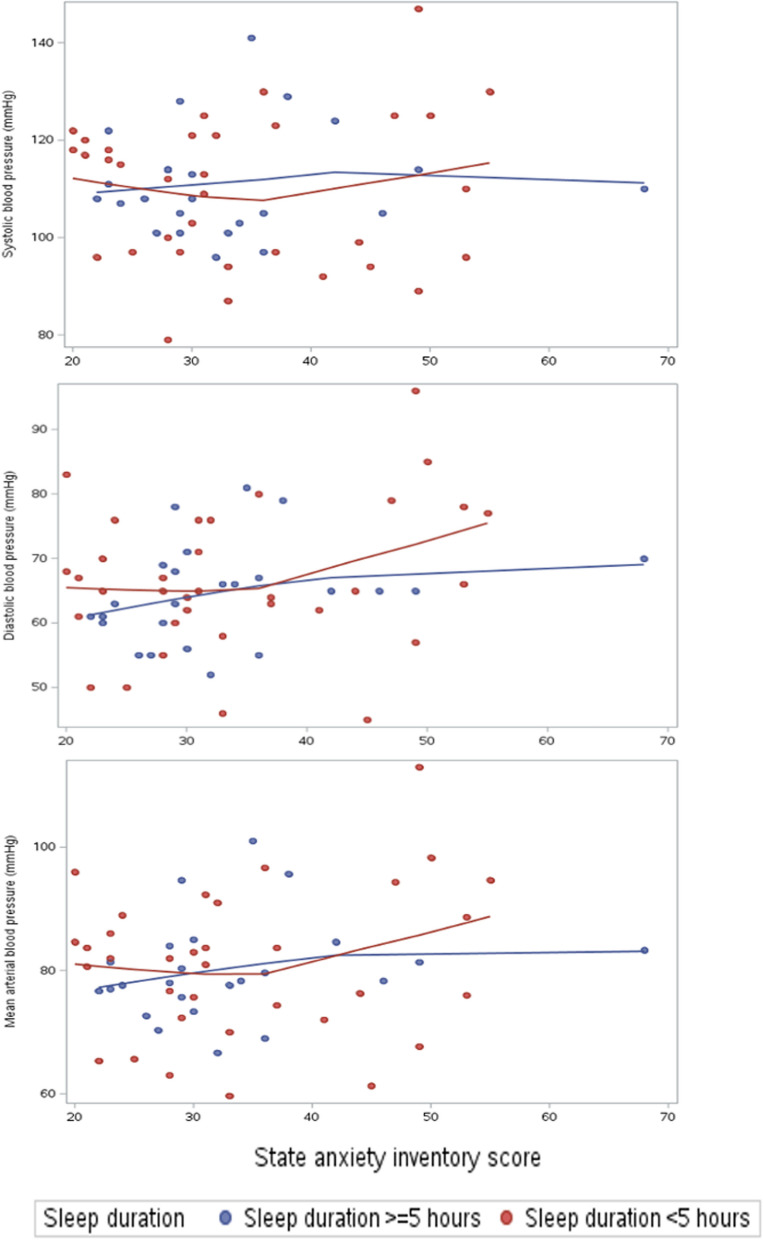
State anxiety inventory score and blood pressure parameter associations stratified by short sleep duration

When short sleep duration was additionally included in Model 3, anxiety was significantly associated with all BP parameters (SBP: β 9.6, *p* = 0.01, DBP: β 8.1, *p* < 0.001, and MAP: β 8.8, *p* < 0.001). Similar to Model 2 results, no significant statistical moderating effect was detected between short sleep duration and anxiety on BP (p-interaction SBP 0.12, DBP 0.24, and MAP 0.13) (Table 2, Fig. 3).

## Discussion

This prospective pilot study demonstrated the degree of blood pressure parameter elevations in pregnant people with anxiety after accounting for sleep disturbances including positive screening for clinical insomnia and objective short sleep duration. Pregnant people with anxiety had 5.1–9.6 mmHg higher systolic, 6.1–8.1 mmHg higher diastolic, and 6.2–8.8 mmHg higher mean arterial pressures than people without anxiety after accounting for potential confounders, including both subjective and objective measures of sleep. There was no evidence of statistical interaction between anxiety and either subjective insomnia or objective short sleep duration on any of the BP parameters. These results highlight the importance of accounting for sleep when studying the relationship between anxiety and blood pressure, as blood pressure elevations were only revealed in models adjusting for sleep.

Previous studies have not simultaneously assessed the potential interplay between anxiety and sleep problems on BP parameters [1–18, 22, 23, 34, 35]. Our results demonstrate that sleep is an important confounder to consider when evaluating the effect of anxiety on blood pressure; in this case, adjusting for sleep revealed an association. Given that small increases in SBP, MAP, and DBP are known to increase cardiovascular risks, our results showing nearly 10 mmHg increases in these parameters with anxiety is alarming. Our results support the prioritization of universal screening and treatment of anxiety as a strategy to reduce maternal morbidity from hypertensive disorders. The impact of insomnia, short sleep duration and other sleep problems on this relationship as well as on overall health outcomes in pregnancy requires further study [14].

This study adds to efforts to identify modifiable risk factors for adverse cardiovascular outcomes in pregnancy [5, 7–14]. A bidirectional relationship between anxiety and sleep problems has previously been described, along with their respective link to hypertensive disorders, yet the specific impact of these potential risk factors, both separately and together, on blood pressure parameters is understudied, especially in pregnancy [4–16, 19, 34]. Our simultaneous examination of anxiety, clinical insomnia, and short sleep duration addresses this knowledge gap. First, consistent with previous studies, we found overlaps between anxiety, insomnia, and short sleep duration in our study [24]. Second, when examining anxiety and blood pressure in pregnancy, we found that anxiety alone was not significantly associated with elevation in blood pressure. This relationship was revealed only in models adjusting for insomnia or short sleep duration, especially in the latter case. Our results suggest that anxiety and sleep are potential modifiable risk factors influencing hypertensive disorders of pregnancy, and therefore interventions to target these specific factors are critically warranted [35].

Given the significant burden that hypertensive disorders pose on maternal morbidity and mortality, identifying potentially modifiable risk factors and initiating timely interventions for these conditions is vital [1–3]. The results of our study demonstrate the effects on blood pressure in pregnant people with two commonly underdiagnosed disorders in pregnancy: anxiety and sleep problems. Anxiety is often underdiagnosed, and screening tools may be underutilized, despite ACOG’s recommendation for anxiety screening during pregnancy and US Preventative Task Force recommendation for universal anxiety screening [36–38]. Healthcare professionals may overlook screening for anxiety, representing a missed opportunity to offer counseling on modifiable risk factors that can impact cardiovascular health. In our study, 25% of the cohort screened positive for anxiety, which is slightly higher than the previously reported 15% in the literature, which we attribute to screening all patients for anxiety using a validated tool that is often underutilized in pregnancy care [11, 36]. Notably, the observed prevalence of clinical insomnia, 18%, was well below the 40–72% national prevalence among pregnant people estimate reported by other studies, which may have reduced power to find any effects of insomnia on the association between anxiety and blood pressure [17]. Similar to previous studies, however, 61% of participants met the criterion for objective short sleep duration, a result that is consistent with increased sleep disturbance during the final trimester of pregnancy. Despite the unexpectedly low incidence of insomnia and high rates of short sleep duration, clinical insomnia but not objective short sleep duration was significantly associated with anxiety. Previous studies are mixed on the strongest associations between anxiety with insomnia and short sleep duration, however in our study the significant association might be explained by anxiety that is focused on perinatal concerns rather than sleep [9].

Our study adds to the growing body of literature on anxiety in pregnancy that demonstrates a relationship between anxiety and individual blood pressure parameters. However, our study also attempted to account for potential additive or interactive effects between anxiety and insomnia or short sleep duration [4–16]. It is interesting that we demonstrated an association between anxiety and blood pressure only after adjusting for sleep disturbances and may be explained by known sleep disturbances activating the hypothalamic–pituitary–adrenal axis resulting in hypertension [13]. A bidirectional relationship between anxiety and sleep problems such as insomnia and short sleep duration has previously been described, and their respective effect on the diagnosis of hypertensive disorders has been demonstrated, yet the specific impact of these potential risk factors, both separately and together, on blood pressure parameters is understudied [4–16]. Interventions to address modifiable risk factors influencing hypertensive disorders of pregnancy, which can serve as markers for adverse lifelong cardiovascular health outcomes, are imperative, highlighting the importance of further investigation of the impact of anxiety, insomnia and short sleep duration as potentially modifiable risk factors for improving overall health outcomes in pregnancy [36, 38]. For example, the benefits of cognitive behavioral therapy based interventions for patients with hypertension have been recognized and can serve as an intervention to address the relationship between mood disorders, sleep problems, and hypertension [39]. Studies are also needed to assess whether interventions that reduce anxiety or sleep disturbance in pregnancy might lower the risk of hypertensive disorders.

Our study has several strengths. We were able to utilize both subjective insomnia and objective total sleep time as data to better understand the impact of sleep on anxiety and blood pressure. We used a validated insomnia questionnaire, recorded objective sleep data using actigraphy, and collected key blood pressure parameters for ascertainment of variables. As our study was prospective, we were able to account for potential confounding factors influencing anxiety, insomnia and short sleep duration, including gestational age, body weight, physical health status, and mental health status [36, 38].

However, our study has several limitations. We included clinical insomnia as a diagnosis, which is suggested by subjective measures of sleep and can be influenced by self-report bias. We could not identify acute or chronic insomnia disorder using criteria from Diagnostic and Statistical Manual of Mental Disorders, 5th Edition, as the study duration was short and we did not use a structured clinical interview, for example, the Structured Clinical Interview for Sleep Disorders [40]. We included participants with chronic hypertension because this is an exploratory study assessing a small cohort, however, we included use of antihypertensives as a proxy for hypertension severity. We did not collect more than one blood pressure measure for each participant, which does not meet rigorous longitudinal, repeated blood pressure measurement for optimally evaluating relationship trends. Inferences from our analyses were hindered by our relatively small sample size, especially when determining the effects of any additive or interactive effects of anxiety and insomnia on BP parameters. Larger prospective studies are needed to better understand how anxiety and sleep disturbance might impact hypertensive disorders of pregnancy and whether interventions could mitigate these effects. Additionally, our study was advertised as a study investigating sleep and anxiety which could ultimately impact those who selected to enroll in the study and subsequent generalizability of the study.

## Conclusions

In our prospective study, when adjusting for insomnia or short sleep duration, state-anxiety was associated with up to a 9.6 mmHg increase in blood pressure parameters. Models adjusting for objective short sleep duration based on Actigraphy data had the largest magnitude of association between anxiety and elevated blood pressure. Our results support the growing movement towards assessing and treating anxiety and sleep problems as a potential strategy to reduce maternal and fetal morbidity from hypertensive disorders [38].

## Data Availability

The dataset analyzed during the current study is available from the corresponding author on reasonable request.

## Abbreviations

ACOG: American College of Obstetricians and Gynecologists
BP: Blood pressure
BMI: Body mass index
DBP: Diastolic blood pressure
GEE: Generalized estimating equations
ISI: Insomnia Severity Index
LPCH: Lucile Packard Children’s Hospital
MAP: Mean arterial pressure
STAI-S: State Trait Anxiety Inventory-State
SBP: Systolic blood pressure
